# Targeting Oncogenic Mutant p53 for Cancer Therapy

**DOI:** 10.3389/fonc.2015.00288

**Published:** 2015-12-21

**Authors:** Alejandro Parrales, Tomoo Iwakuma

**Affiliations:** ^1^Department of Cancer Biology, University of Kansas Medical Center, Kansas City, KS, USA

**Keywords:** mutant p53, depletion, compounds, reactivation, cancer therapy, gain of function, dominant negative, oncogenes

## Abstract

Among genetic alterations in human cancers, mutations in the tumor suppressor *p53* gene are the most common, occurring in over 50% of human cancers. The majority of p53 mutations are missense mutations and result in the accumulation of dysfunctional p53 protein in tumors. These mutants frequently have oncogenic gain-of-function activities and exacerbate malignant properties of cancer cells, such as metastasis and drug resistance. Increasing evidence reveals that stabilization of mutant p53 in tumors is crucial for its oncogenic activities, while depletion of mutant p53 attenuates malignant properties of cancer cells. Thus, mutant p53 is an attractive druggable target for cancer therapy. Different approaches have been taken to develop small-molecule compounds that specifically target mutant p53. These include compounds that restore wild-type conformation and transcriptional activity of mutant p53, induce depletion of mutant p53, inhibit downstream pathways of oncogenic mutant p53, and induce synthetic lethality to mutant p53. In this review article, we comprehensively discuss the current strategies targeting oncogenic mutant p53 in cancers, with special focus on compounds that restore wild-type p53 transcriptional activity of mutant p53 and those reducing mutant p53 levels.

## Introduction

The tumor suppressor p53 exerts its biological function by regulating transcription of numerous downstream target genes involved in cell cycle arrest, apoptosis, DNA repair, senescence, and metabolism as a transcription factor (1, 2). p53 is also directly recruited to the mitochondria and induces apoptosis independent of its function as a transcription factor (3). Under unstressed physiological conditions, p53 expression is maintained at a low level, mainly by being degraded by its E3 ubiquitin ligases, MDM2, Pirh2, and COP1 (4). Once cells are exposed to genotoxic stresses, p53 is posttranslationally modified through phosphorylation and acetylation, becomes stabilized, and induces cell cycle arrest and/or cell death. When p53 activity is lost by gene deletion or mutations, normal cells lose the abilities to control their growth and death, leading to immortalization and ultimately cancer (5). The observation that over 50% of human cancers have mutations in the *p53* gene indicates the indispensability of intact p53 activity for suppressing tumor development (6).

Mutations in the *p53* gene occur mainly in the DNA-binding domain, the majority of which are missense mutations, resulting in loss of function as a transcription factor and accumulation of dysfunctional p53 protein in tumors (7). Mutant p53 can be categorized roughly into two types: DNA contact (class I) mutant where mutations are present on amino acids directly binding to the p53-responsive element in DNA (e.g., p53^R273H^ and p53^R280K^) and conformational (class II) mutant in which mutations alter structure of p53 to abolish its DNA-binding ability (e.g., p53^R175H^ and p53^V143A^) (8). Both the mutant types not only lose the transcriptional activity, but also have the dominant-negative (DN) activity by hetero-oligomerizing with wild-type p53. Moreover, mutant p53 shows oncogenic gain-of-function (GOF) activities, such as enhanced tumor progression, metastatic potential, and drug resistance, when overexpressed even in cells lacking wild-type p53 (7). These findings are supported by the fact that p53 was originally appreciated as an oncogene, since researchers unknowingly used plasmids encoding mutations in the *p53* gene. Thus, mutant p53 functions as an oncogene and greatly contributes to malignant properties of cancer cells.

Disrupting specific mechanisms which cancer cells develop for their survival and growth is a rational approach to selectively kill cancer cells with minimal effects on normal cells. In this regard, mutant p53 is one of the best druggable targets, since over half of human cancers have p53 mutations, while normal cells mostly do not have mutations in the *p53* gene (9). To exploit the frequent presence of mutant p53 in tumors and target mutant p53 in cancer therapy, two strategies including restoration of wild-type p53 transcriptional activity and depletion of mutant p53 have been extensively undertaken, in addition to inhibition of downstream target pathways involved in mutant p53 GOF and induction of synthetic lethality to mutant p53. Since mutant p53 is generally accumulated in tumors (10), reactivating p53 activity can efficiently induce proliferation arrest and/or cell death of cancer cell. Specifically, in the late stage of tumor development, cancer cells express only mutant p53 with loss of heterozygosity of the other wild-type *p53* allele (11, 12). Such cells often have high metastatic and chemotherapy resistant properties. Hence, this p53 reactivation strategy is powerful to treat cancers expressing mutant p53. The other strategy to specifically deplete oncogenic mutant p53 in cancer cells should have minimal impact on wild-type p53, since depletion of wild-type p53 in normal and cancer cells can accelerate tumorigenesis or tumor progression. Accumulating studies suggest that knockdown of mutant p53 significantly reduces oncogenic potential of cancer cells expressing only mutant p53 (13–16), suggesting that malignant properties of cancer cells are, at least partially, dependent on the presence of mutant p53. This could be simply due to the loss of oncogenic activity of mutant p53 or possibly because cancer cells are addicted to mutant p53 for their survival and proliferation. This strategy would work even better when cancer cells retain the wild-type *p53* allele with the mutant *p53* allele (heterozygous), since it can also restore wild-type p53 activity which is suppressed by the DN activity of mutant p53. Thus, depletion of mutant p53 is also an effective strategy to suppress tumor progression.

In this article, toward developing precision cancer medicine, we summarize updated information about compounds which can restore wild-type p53 activity, as well as those depleting mutant p53.

## Drugs/Compounds That Restore Wild-Type p53 Activity

Most p53 mutants lose their ability to bind with p53-response elements in DNA, thereby losing transcriptional activity and tumor suppressive function (17). However, the following evidence suggests that sequence-specific p53 transcriptional activities can be restored from mutant p53: (1) many p53 mutants are temperature sensitive and restore the p53 activity at the permissive temperature (18, 19), (2) synthetic peptides, CDB3 and Peptide 46 which are derived from 53BP2 and C-terminal domain of p53, respectively, restore the sequence-specific DNA binding and transcriptional activity of p53 (20, 21), and (3) insertion of second-site mutations or a N-terminal deletion in several p53 mutants restore the p53 transcriptional activities (22–24). Since the first p53-reactivating compound, CP-31398, was identified (17), investigators have made tremendous efforts to identify compounds that restore p53 transcriptional activity. Major compounds related to mutant p53 reactivation are listed in Table 1 and explained below.

**Table 1:** **Compounds that induce reactivation of mutant p53**.

Compound	Type of mutant	Mechanism	Reference	Structure
CP-31398	V173A, S241F, R249S, R273H	Stabilize the DNA-binding core domain and induce conformational change	(17, 25–27)	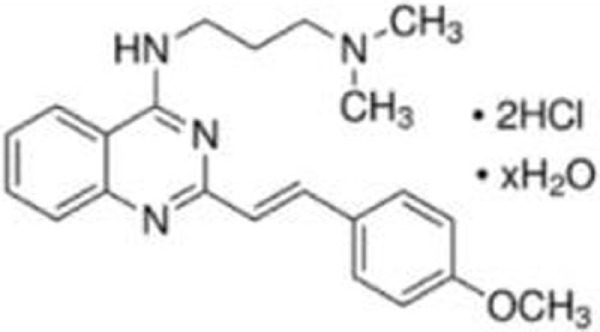
STIMA-1, structural similarity to CP-31398	R175H, R273H	Bind to the cysteine residues in the core domain and stabilize wild-type p53 conformation	(28)	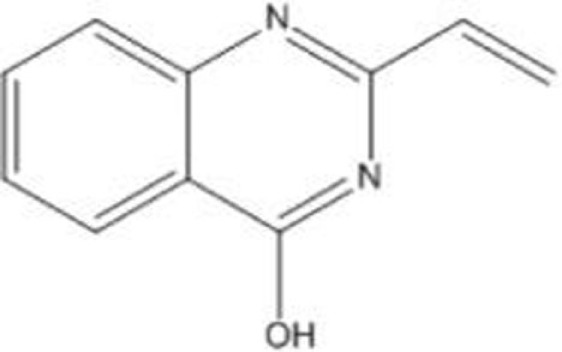
PRIMA-1 and the methylated analog (APR-246/PRIMA-1^MET^)	R175H, R273H	Bind to thiol groups in the core domain and restore wild-type conformation	(23, 29, 30)	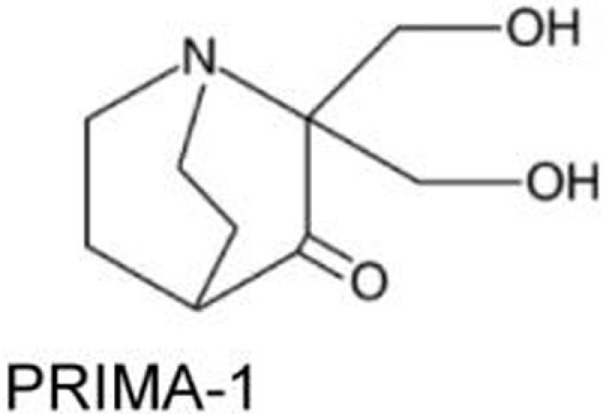
MIRA-1 (NSC19630), and its analogs MIRA-2 and -3	R175H, R248Q, R273H	Prevent unfolding of wild-type and mutant p53 and restore native wild-type p53 conformation	(31)	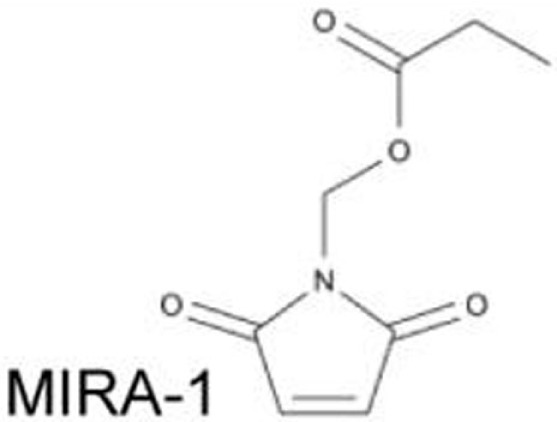
RITA (NSC652287)	R175H, R248W, R273H, R280K	Restore p53 transcriptional activity and induce apoptosis	(32, 33)	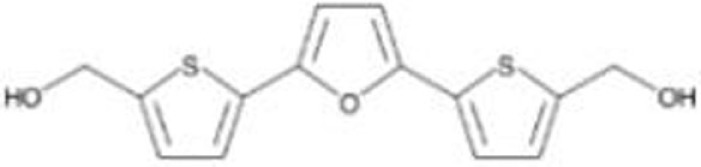
NSC319726/ZMC 1 (zinc metallochaperone-1)	R175H, R172H (mouse)	Restore wild-type p53 conformation and activity with MDM2-dependent degradation	(34–36)	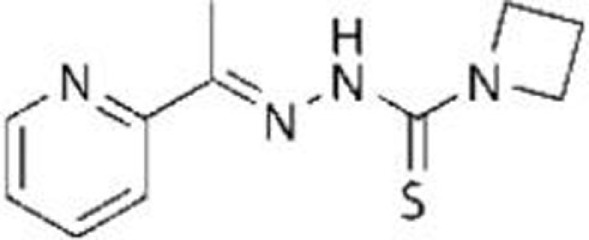
Chetomin (CTM)	R175H	Increase Hsp40 (DNAJB1) levels and Hsp40-p53^R175H^ binding, restoring wild-type p53 conformation, activity, and MDM2-dependent degradation	(37)	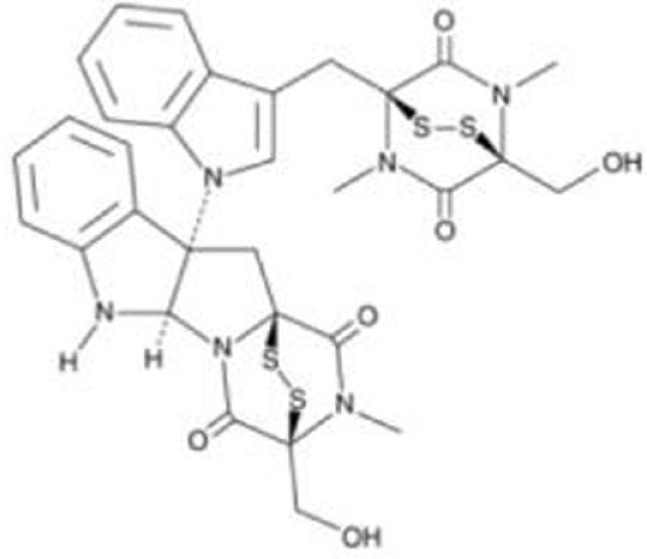
PK7088	Y220C	Bind to a p53^Y220C^-specific surface cavity and stabilize p53^Y220C^ with restored wild-type p53 conformation	(38)	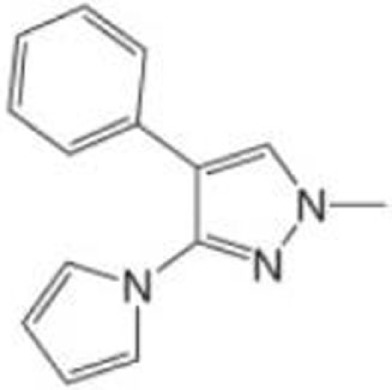
Stictic acid (NSC87511)	R175H, G245S	Target cysteine 124 at the p53 core domain and restore wild-type p53 activity	(39)	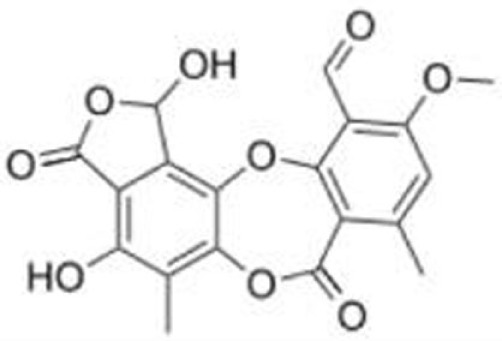
p53R3	R175H, M237I, R273H	Restore sequence-specific DNA binding and p53 transcriptional activities	(40)	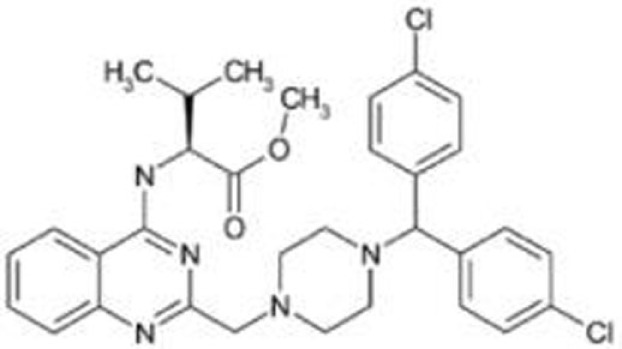
SCH529074	R175H, L194F, R248W, R249S, R273H	Restore sequence-specific DNA binding and p53 transcriptional activities	(41)	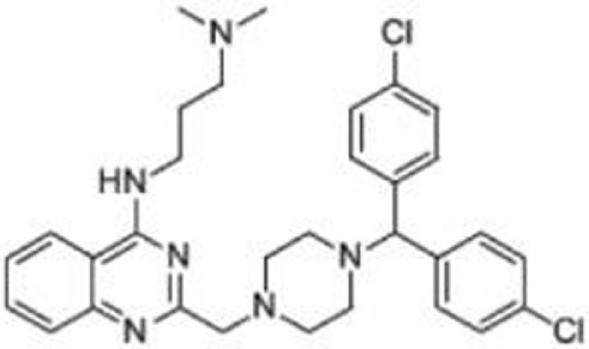
WR-1065	V272M	Restore DNA binding and transcriptional activities of p53^V272M^	(42–45)	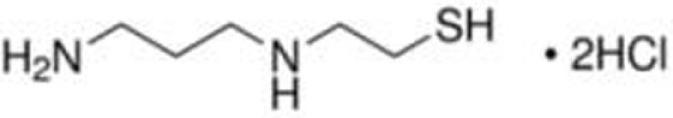

### CP-31398

CP-31398 (styrylquinazoline) was identified through a structure-based screening as a compound which could restore native wild-type p53 conformation from a denatured conformation in the DNA-binding domain, using a conformation specific antibody PAb1620. CP-31398 leads to increase in *p21* mRNA expression in Saos-2 (p53-null) cells expressing p53^V173A^ and p53^R249S^ mutants, and inhibits tumor growth of A375.S2 (p53^R249S^) and DLD1 (p53^S241F^) cells (17). CP-31398 increases mRNA expression of *MDM2* and *p21* in multiple cancer cell lines (46). CP-31398 also induces mitochondrial translocation of mutant p53^R273H^ in A431 skin carcinoma cell line, leading to cytochrome c release and apoptosis (25). Intriguingly, CP-31398 cannot refold already misfolded mutant p53 proteins, since cycloheximide prevents the effect of CP-31398 on p53 restoration (23, 26). It also induces cell death in a p53-independent manner through free radical formation (27).

### STIMA-1 (SH Group-Targeting Compound That Induces Massive Apoptosis)

STIMA-1 [2-vinylquinazolin-4-(3H)-one] was identified as one of the CP-31398 derivatives, which induced mutant p53 (p53^R175H^ and p53^R273H^)-dependent growth suppression (28). Both CP-31398 and STIMA-1 bind to the cysteine residues in the core domain of mutant p53, leading to stabilization of wild-type p53 conformation and subsequent restoration of transcriptional activity (28). STIMA-1 increases the DNA-binding ability of mutant p53, resulting in upregulation mRNA expression of *p21*, *PUMA*, and *BAX*, and leading to mutant p53-dependent apoptosis (28).

### PRIMA-1 and PRIMA-1^MET^/APR-246

PRIMA-1 [2,2-bis (hydroxymethyl)-3-quinuclidinone] was identified through a screening as a compound that suppressed proliferation of Saos-2 osteosarcoma cell line expressing p53^R273H^ (Saos-2-p53^R273H^) with little effect on the parental Saos-2 cells. PRIMA-1 and its methylated analog PRIMA-1^MET^ (also known as APR-246) not only enhance stability of wild-type p53 at 37°C, but also induce conformational change of p53^R175H^, leading to restoration of DNA-binding activity of p53^R175H^ with increased *MDM2* and *p21* mRNA expression (23). Notably, PRIMA-1 refolds previously accumulated unfolded mutant p53 (23). The mechanisms underlying refolding of mutant p53 by PRIMA-1 and PRIMA-1^MET^ involve the conversion of these compounds to products which form adducts with thiol groups in the mutant p53 core domain, leading to restoration of wild-type conformation and induction of apoptosis in tumor cells (29, 30). Several studies have successfully validated their tumor suppressive effects in mouse models of multiple types of cancer (47–50). Importantly, PRIMA-1^MET^ is currently in clinical trials (51, 52).

### MIRA-1 and Its Structural Analogs

Using the same screening strategy as PRIMA-1, MIRA-1 (NSC19630) was identified as a compound that suppressed proliferation of Saos-2-p53^R273H^ cells (31). MIRA-1 and its structural analogs MIRA-2 and MIRA-3 from the NCI repository inhibit proliferation of cancer cells expressing p53^R175H^ and p53^R273H^ (31). Both MIRA-1 and MIRA-3 also induce cell death in cancer cells expressing mutant p53 (31). Furthermore, MIRA analogs prevent unfolding of wild-type and mutant p53, and also restored native wild-type p53 conformation, leading to enhanced DNA-binding activity of mutant p53 (p53^R175H^ and p53^R248Q^) and increase in mRNA expression of p53 downstream target genes, *MDM2* and *p21*, in several mutant p53-carrying cancer cell lines (31). *In vivo* effects of MIRA analogs have also been confirmed in mouse models (31, 53).

### RITA (NSC652287)

NSC652287 [2,5-bis(5-hydroxymethyl-2-thienyl) Furan] is one of a series of thiophene derivatives and is known to inhibit tumor growth of renal cell carcinoma cells with DNA–protein cross-linking and upregulation of wild-type p53 and p21 (54, 55). NSC652287 (RITA: reactivation of p53 and induction of tumor cell apoptosis) is also identified through cell proliferation assay-based screening using isogenic cell lines of HCT116 (wild-type p53 and p53-null) as a compound that suppresses the growth of HCT116 (wild-type p53) cells in a dose-dependent manner with minimum effects on HCT116 (p53-null) cells (56). Later, NSC652287/RITA was found to suppress the growth of cancer cell lines carrying various p53 mutants (p53^R175H^, p53^R213Q/Y234H^, p53^R248W^, p53^R248Q^, p53^I254D^, p53^R273H^, and p53^R280K^) by restoration of p53 transcriptional activity (*p21*, *NOXA*, *PUMA*, and *GADD45*) and induction of apoptosis through upregulation of pro-apoptotic proteins and downregulation of several oncogenes or anti-apoptotic proteins (32, 33, 57). However, the exact mechanism by which RITA activates both wild-type and mutant p53 to induce apoptosis remains unclear.

### NSC319726/ZMC1 (Zinc Metallochaperone-1)

Zinc is required for proper folding of p53 protein, while lack of zinc in the central core domain of p53 leads to unfolding (23, 58, 59). Also, addition of zinc to cells or its administration to mice carrying tumors are known to restore DNA-binding activity of mutant p53 (p53^R175H^ and p53^R273H^) in cells and tumors, leading to tumor suppression (60). Thus, facilitating the binding of mutant p53 to zinc can be used to restore the proper folding and transcriptional activity of mutant p53 (34). Indeed, NSC319726 [zinc metallochaperone-1 (ZMC1)], a thiosemicarbazone derivative, was identified in a screen of the NCI60 panel of human tumor cell lines as a compound that exhibited selective toxicity to cells carrying p53^R175H^ with minimum effects on cells expressing wild-type p53 and other p53 mutants (p53^R248Q^ and p53^R273H^) (35). NSC319726 restores the wild-type-like conformation of mutant p53 and upregulates p53 downstream target genes (*p21*, *PUMA*, and *MDM2*) through increasing ROS levels (35, 36). It also reduces p53^R175H^ levels, which is rescued by Nutlin-3a (35). Importantly, its administration induces greater toxicity in *p53^*R172H/R172H*^* (equivalent to human p53^R175H^) mice than in wild-type mice in a dose-dependent manner (35). NSC319726 also suppresses tumor growth of TOV112D (p53^R175H^) cells *in vivo* (35). Other thiosemicarbazone family members (NSC319726 and NSC328784) also preferentially reduce cell viability of p53 mutant cell lines (35).

### Chetomin

Hiraki et al. (37) performed high-throughput chemical library screening using a luciferase reporter with the p53 response element of the *PUMA* promoter in H1299 (p53-null) cells carrying p53^R175H^, which identified chetomin (CTM) as a compound that increased luciferase activity dose-dependently. CTM suppresses cancer cell growth *in vitro* and *in vivo* more efficiently in cells expressing p53^R175H^ with upregulation of MDM2, p21, and PUMA than those expressing wild-type p53 and p53^R273H^, as well as null for p53 (37). The effects of CTM on reduced p53^R175H^ levels are dependent on MDM2 (37). Interestingly, CTM increases protein levels of Hsp40 (DNAJB1) and the binding of p53^R175H^ to Hsp40, leading to restoration of wild-type p53 conformation and tumor suppression of cancer cells carrying p53^R175H^ (37). However, CTM is also known to inhibit the hypoxia-inducible factor pathway by blocking the interaction of HIF proteins and their cofactor p300. Moreover, it suppresses *in vivo* tumor growth of HCT116 (wild-type p53) cells and enhances radiosensitivity of cancer cells regardless of the p53 status (61–63). Thus, CTM has other function than mutant p53 reactivation.

### PK7088

PK7088 [1-methyl-4-phenyl-3-(1H-pyrrol-1-yl)-1H-pyrazole] was identified as a compound that binds to a p53^Y220C^-specific surface cavity destabilizing this protein through protein-observed NMR screening (38, 64). PK7088 stabilizes p53^Y220C^ and restores wild-type p53 conformation. It is biologically active in cancer cells carrying p53^Y220C^ mutant and induces G2/M arrest of the cell cycle and apoptosis with upregulation of NOXA and p21, as well as relocation of BAX to the mitochondria (38). PK7088 and Nutlin-3a cooperatively upregulate protein expression of p21 and NOXA (38). Crystal structure of p53-Y220C core domain with PK7242, a more soluble PK7088 analog, is also presented (38).

### Stictic Acid (NSC87511)

A computational analysis of p53 structural models suggests that cysteine 124 of p53 is located at the center of a transiently open binding pocket between loop L1 and sheet S3 of the p53 core domain (39). Based on the finding that additional mutation at cysteine 124 to alanine on p53^R175H^ (p53^C124A/R175H^) abolished the effects of PRIMA-1 on the reactivation of p53^R175H^, Wassman et al. (39) performed an Ensemble-based virtual screening against this pocket and identified stictic acid as a p53 reactivation compound (39). Stictic acid stabilizes p53 *in vitro* and induces expression of p21 and PUMA in Saos2 (p53-null) cells expressing mutant p53 (p53^R175H^ and p53^G245S^) (39).

### P53R3

The p53 reactivator (P53R3) is a compound identified through a screening using an *in vitro* DNA-binding assay (40). P53R3 restores sequence-specific DNA binding of several p53 mutants (p53^R175H^, p53^M237I^, and p53^R273H^) and induces p53-dependent antiproliferative effects with increase in mRNA expression of many p53 target genes, such as *p21*, *GADD45*, *BAX*, *PUMA*, *PIG3*, and *MDM2* (40). It should be noted that P53R3 also increases mRNA expression of several p53 target genes (*p21*, *GADD45*, *PUMA*, and *MDM2*) in cancer cells expressing wild-type p53 (40).

### SCH529074

The small molecule SCH529074 was identified by a DNA-binding assay-based screening as a compound that enabled p53^R273H^ to bind to a consensus p53 DNA-binding site (41). SCH529074 restores the PAb1620 epitope by acting as a chaperone and enhances DNA-binding activity of several p53 mutants (p53^R175H^, p53^S241F^, p53^R248W^, p53^R249S^, and p53^R273H^), leading to upregulation of p53 downstream target genes (*p21*, *BAX*, *NOXA*, *cyclin G1*, and *PUMA*), induction of proliferation arrest or apoptosis, and inhibition of *in vivo* tumor growth of mutant p53-expressing cell lines (41). Additionally, SCH529074 binds to DNA-binding domain of p53 and inhibits ubiquitination and degradation of wild-type p53 by MDM2 (41).

### WR-1065

WR1065 is an active form of amifostine and is used to protect tissues against the damaging effects of radiation and chemotherapeutic drugs (42). WR-1065 increases wild-type p53 activity through a JNK-dependent signaling pathway, but not through genotoxic mechanisms (42–44). It is also reported that WR-1065 restores wild-type p53 conformation of a temperature-sensitive p53^V272M^, leading to increase in the DNA-binding activity, transactivation of several p53 target genes (*p21*, *GADD45*, and *MDM2*), and cell cycle arrest in G1 phase (45).

## Compounds That Deplete Mutant p53

Although many p53-reactivating compounds seem to target more than one p53 mutant, it remains unclear if these compounds can reactivate all p53 mutants or specific mutant types. Also, PRIMA-1^MET^ is the only drug currently under clinical trials. Thus, the development of p53-reactivating compounds remains challenging. Another approach to target oncogenic mutant p53 is to discover compounds that specifically deplete mutant p53 with little effect on wild-type p53. Rationale to develop mutant p53-depleting compounds is based on the following observations: (1) mutant p53 is inherently unstable, but once stabilized, it can accelerate tumor progression (10) and (2) knockdown of mutant p53 by small interference RNAs (siRNAs) or shRNAs reduces malignant properties of cancer cells (13–16), thus indicating that oncogenic potential of cancer cells are, at least partially, dependent on the presence of accumulated oncogenic mutant p53. Although the mechanisms behind stabilization or degradation of mutant p53 are not necessarily the same as those of wild-type p53 and remain elusive (5, 65), several compounds that induce mutant p53 degradation without altering wild-type p53 have been found. These compounds can be used as effective therapeutic strategies for both cancers carrying only mutant p53 and those retaining wild-type p53 with mutant p53 as mentioned in the Introduction. Thus, compounds that specifically deplete mutant p53 are valuable for cancer therapy and also for elucidating the mechanisms of stabilization or degradation of mutant p53. Major compounds which deplete mutant p53 are listed in Table 2 and explained below.

**Table 2:** **Compounds that deplete mutant p53**.

Compound	Type of mutant	Mechanism	Reference	Structure
Hsp90 inhibitors: 17-AAG, geldanamycin, ganetespib	R175H, L194F, R248Q, R273H, R280K, R172H (mouse)	Reverse the Hsp90’s function to inactivate MDM2 and CHIP	(13, 66–69)	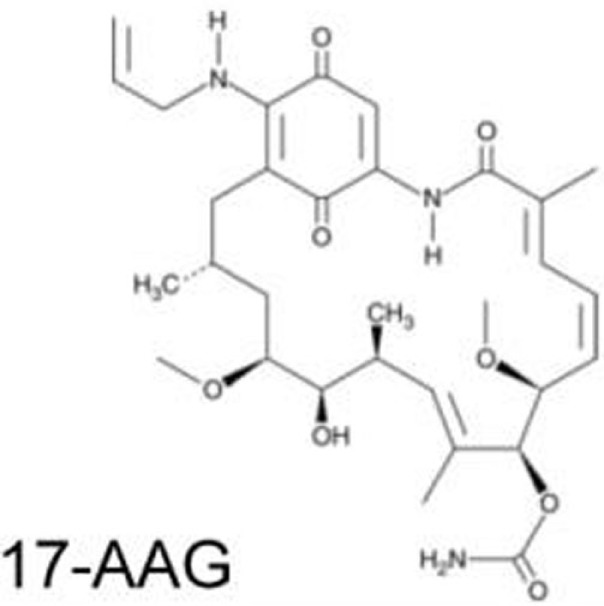
HDAC inhibitors: vorinostat/SAHA, romidepsin/depsipeptide	R175H, R280K, V247F/P223L	Inhibit HDAC6 and disrupt the HDAC6/Hsp90/mutant p53 complex	(70–72)	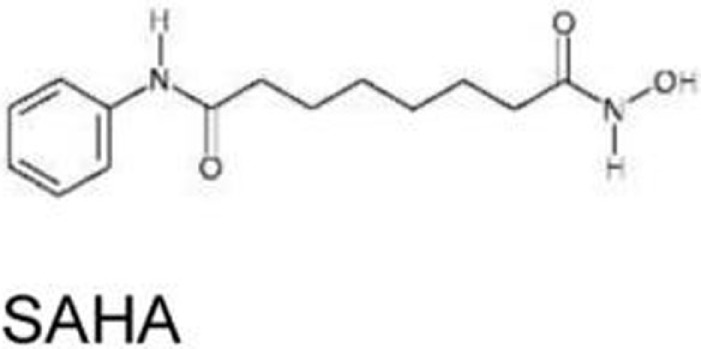
Arsenic compounds	R175H, R248W, H179Y/R282W, R273H	Increase transcripts of Pirh2 and induce degradation of mutant p53	(73, 74)	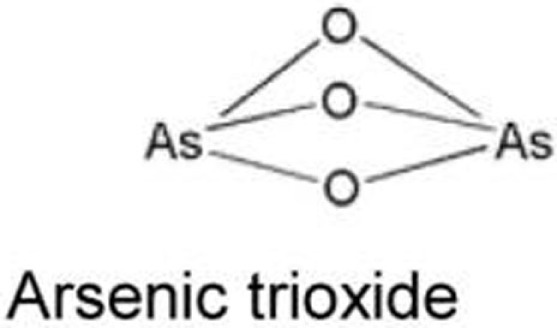
Gambogic acid	R175H, G266E, R273H, R280K	Inhibit the mutant p53-Hsp90 complex and induce CHIP-dependent degradation	(75)	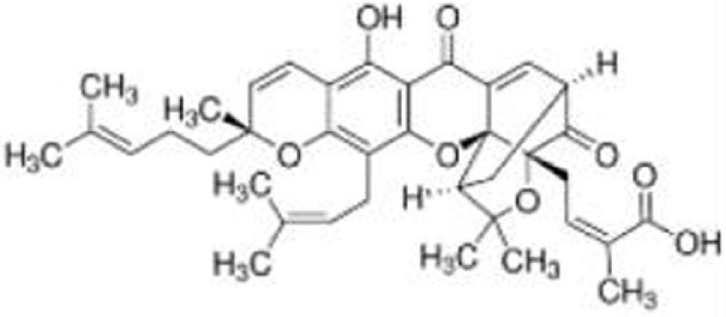
Spautin-1	R158lnF, R175H, S241F, R248Q, G266Q, R280L, R273H	Induce mutant p53 degradation via the CMA pathway activated by the suppression of macroautophagy under glucose-free and confluent conditions	(76, 77)	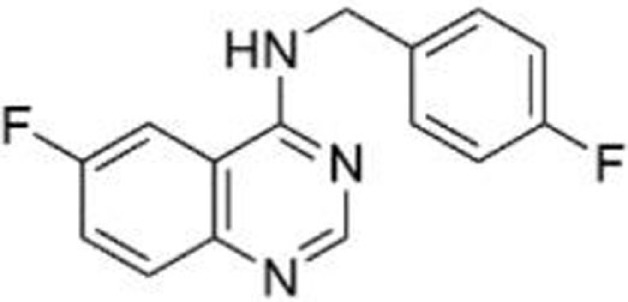
YK-3-237	V157F, M237I, R249S, R273H, R280K	Decrease mutant p53 levels through deacetylation at lysine 382 by activating SIRT1	(78)	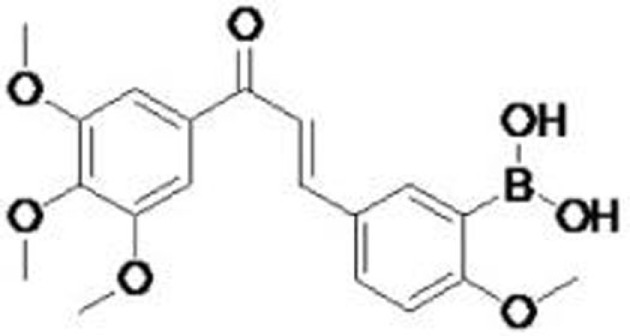
NSC59984	R175L, R175H, S241F, R273H/P309F	Induce MDM2-mediated mutant p53 degradation and activate p73	(79)	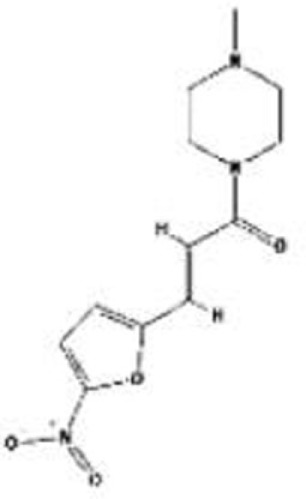
Disulfiram (DSF)	R273H	Induce degradation of both wild-type p53 and p53^R273H^ via the 26S proteasome pathway	(80)	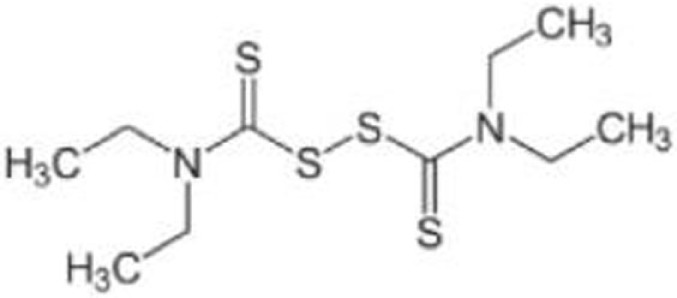

### Hsp90 Inhibitors: Geldanamycin, 17-AAG, Ganetespib

Blocking the function of heat shock protein 90 (Hsp90) leads to depletion of several oncogenic proteins, such as Raf-1, ErbB2, and mutant p53 (66, 67), because Hsp90 contributes to accumulation of mutant p53 by inactivating p53 ubiquitin ligases, MDM2, and CHIP (68, 69). Treatment of cancer cells with 17-AAG, a Hsp90 inhibitor and an analog of geldanamycin, promotes degradation of varieties of p53 mutants (p53^R175H^, p53^L194F^, p53^R273H^, and p53^R280K^) and decreases viability of cells carrying mutant p53 (69). Importantly, another Hsp90 inhibitor, ganestespib, which is 50-fold more potent than 17-AAG in destabilizing mutant p53 with little effect on wild-type p53 levels, induces mutant p53 depletion with increased apoptosis in tumors *in vivo* in both p53^R248Q^ Hupki (human p53 knock-in) and p53^R172H^ knock-in mouse models (13). Ganetespib is currently under evaluation in clinical trials, including phase III for lung cancer (81–83).

### Histone Deacetylase Inhibitors: Vorinostat/SAHA, Romidepsin/Depsipeptide

The effects of histone deacetylase inhibitors (HDACi) on mutant p53 (p53^R175H^, p53^R280K^, and p53^V274F/P223L^) were first reported by Blagosklonny et al. (70). Later, two mechanisms describing the inhibition of HDAC8-mediated mutant p53 transcription (84) and mutant p53 destabilization through inhibition of HDAC6 by HDACi are proposed (71). Specifically, suberoylanilide hydroxamic acid (SAHA, also known as vorinostat), a FDA-approved HDACi that inhibits class I, II, and IV HDACs, induces degradation of mutant p53 by inhibiting HDAC6 activity, an essential positive regulator of Hsp90, and subsequent disruption of the HDAC6/Hsp90/mutant p53 complex, leading to mutant p53 ubiquitination by MDM2 and CHIP (71, 72). SAHA shows higher cytotoxic effects on cancer cells carrying mutant p53 than those having wild type or null for p53 (72). SAHA also sensitizes cancer cells to a topoisomerase inhibitor camptothecin in a mutant p53-dependent manner (71).

### Arsenic Compounds

Arsenic trioxide (ATO), which is used to treat patients with acute promyelocytic leukemia (APL), binds to thiol groups in cysteine residues and induces degradation of proteins, such as promyelocytic leukemia protein (PML) and PML-retinoic acid receptor α (PML-RARα) fusion protein (85). It also activates wild-type p53 and upregulates p53 downstream target genes with induction of apoptosis (86). Yan et al. (73) asked the possibility of using arsenic compounds to target mutant p53 for degradation and found that ATO or sodium arsenite induced proteasomal-dependent degradation of several p53 mutants (p53^R175H^, p53^H179Y/R282W^, p53^R248W^, and p53^R273H^). ATO also increases transcripts of an E3 ubiquitin ligase Pirh2, leading to ubiquitination and degradation of several mutant p53 (74). However, it should be noted that arsenic compounds have carcinogenic effects and are known to induce several types of cancer (87).

### Gambogic Acid

Gambogic acid (GA), a natural product derived from Garcinia hanburyi tree, induces apoptosis and inhibits tumor growth *in vivo* by upregulating wild-type p53 at protein levels (88). On the other hand, GA induces nuclear exports of mutant p53 (p53^R175H^, p53^G266E^, p53^R273H^, and p53^R280K^) for degradation by CHIP ubiquitin ligase (75). GA prevents the mutant p53-Hsp90 complex formation but enhances the mutant p53-Hsp70 interaction (75). Biologically, GA reduces viability of mutant p53-expressing cancer cells and also increases cytotoxic effects of several chemotherapy drugs in human breast cancer MDA-MB-435 (p53^G266E^) cell line (75).

### Spautin-1

Spautin-1 is a derivative of MBCQ (4-((3,4-methylenedioxybenzyl)amino)-6-chloroquinazoline) which was identified as a small molecule that inhibited autophagy through an imaging-based screen using LC3-GFP as a marker (89). When cancer cells are placed in the nutrient-deficient environment, cells start autophagy to generate an alternative energy source to survive by degrading cellular proteins and organelles in the lysosome. Spautin-1 inhibits ubiquitin-specific peptidases, USP10 and USP13, and promotes the degradation of Vps34-PI3 kinase complexes, key regulators of autophagy, leading to inhibition of autophagy (89). Since USP10 also deubiquitinates wild-type p53, Spautin-1 promotes degradation of wild-type p53 (89, 90). Moreover, suppression of macroautophagy by Spautin-1 under glucose-free and confluent conditions is found to induce degradation of several p53 mutants (p53^R158InF^, p53R175H, p53^R248Q^, p53^S241F^, p53^G266E^, p53^R280L^, and p53^R273H^) through the chaperone-mediated autophagy (CMA) pathway (76). Spautin-1 also induces cell death under non-proliferating condition only when cancer cells express mutant p53. This effect of Spautin-1 is independent of MDM2 and the ubiquitin–proteasome pathway, but is dependent on nuclear export of mutant p53 and the presence of Hsc70 (76, 77).

### YK-3-237

YK-3-237 was originally identified as a compound that showed antiproliferative effects in different cancer cell lines, but its mechanism of action was unknown (91). Yi et al. (78) investigated the effects of this compound on the proliferation of breast cancer cell lines carrying different p53 status and found that YK-3-237 preferentially inhibited the proliferation of breast cancer cell lines carrying mutant p53. YK-3-237 also decreased the levels of mutant p53 (p53^V157F^, p53^M237I^, p53^R249S^, p53^R273H^, and p53^R280K^) through reduction in acetylation at lysine 382 (K382) of mutant p53, a target site of a NAD^+^-dependent protein deacetylase SIRT1 (also known as sirtuin 1) (78). Indeed, YK-3-237 activates SIRT1 enzyme activity (78). Furthermore, treatment of triple-negative breast cancer cell lines with YK-3-237 results in induction of apoptosis and G2/M arrest of the cell cycle with increase in mRNA expression of *NOXA* and *PUMA* (78). However, the underlying mechanism remains unclear.

### NSC59984

The small molecule NSC59984 was recently identified as a compound that increased luciferase activity in SW480 (p53^R273H/P309S^) and DLD-1 (p53^S241F^) cells carrying a p53-responsive luciferase reporter construct (PG13) (79). NSC59984 increases mRNA and protein levels of several p53 targets, such as p21, PUMA, and NOXA, with increase in apoptosis. It should be noted that NSC59984 also induces apoptosis in cancer cells having wild type and null for p53, suggesting it has p53-independent effects on cell death (79). Moreover, NSC59984 induces degradation of several p53 mutants (p53^R175L^, p53^R175H^, p53^S241F^, and p53^R273H/P309S^) through MDM2-mediated ubiquitination, whereas it rather increases wild-type p53 levels (79). Importantly, the effects of NSC59984 on p53 target gene expression and apoptosis are caused by activation of p73, rather than conformational changes of mutant p53 (79). In *in vivo* xenograft mouse models, NSC59984 suppresses tumor growth of DLD-1 in a p73-dependent manner (79).

### Disulfiram

Disulfiram (DSF) is used for the treatment of chronic alcoholism by inhibiting acetaldehyde dehydrogenase. DSF has also been under clinical trials for some types of cancer including glioblastoma multiforme and metastatic non-small cell lung cancer (92, 93). DSF and its metabolites which are strong ROS inducers contain a reactive disulfide bond and readily mediate thiol-conjugating reactions, leading to S-glutathionylation of cysteine residues in proteins (94, 95). Protein S-glutathionylation in response to oxidative stress can affect function and stability of target proteins (80, 96). Interestingly, p53 is found to be S-glutathionylated at cysteine residues 124 and 141 (97, 98). Paranjpe et al. (80) reported that DSF and its derivative copper-chelated disulfiram (CuDSF) induced degradation of both wild-type p53 and p53^R273H^ through the 26S proteasome pathway. However, DSF-induced protein degradation is not p53-specific, because it also induces degradation of other redox-regulated proteins, such as NF-κB subunit p50 and UBE1 (80).

## Other Strategies to Target Mutant p53 and Its Related GOF Activity

### Compounds That Induce Readthrough of Premature Termination Codons

About 8% of all the p53 mutations in human cancers are nonsense mutations, which results in the presence of premature termination codons (PTCs) (99). PTC leads to the mRNA degradation by the nonsense-mediated mRNA decay pathway or potential production of truncated proteins. However, aminoglycoside antibiotics (gentamicin, G418, and amikamicin) bind with the ribosomal RNA and promote readthrough of PTCs, leading to partial restoration of full-length protein production (100). Specifically, gentamicin, G418, and NMDI14 induce production of full-length functional p53 from p53^Q192X^, p53^R213X^, and p53^E298X^, leading to apoptosis induction in cancer cells carrying nonsense p53 mutations (101, 102).

### Knockdown with Small Interference RNA

Knocking down specific protein expression by siRNAs or shRNAs can be a specific and potent strategy to target cancers if methodologies of efficient *in vivo* delivery are established (103). Indeed, downregulation of mutant p53 in T47D (p53^L194F^), MDA-MB-231 (p53^R280K^), and MDA-MB-468 (p53^R273H^) breast cancer cell lines induces PARP-dependent apoptosis (104, 105). In DU145 (p53^P223L^/^V274F^) prostate cancer cell line, downregulation of p53^P223L^/^V274F^ by siRNA induces cell cycle arrest at G1 and G2/M phases, as well as apoptosis in a PI3K/Akt-dependent manner (106). Also, silencing of mutant p53 in 5637 (p53^R280T^) and T24 (in-frame deletion of Y126) bladder cancer cell lines by *p53* siRNA induces G2 arrest of the cell cycle and apoptosis, and increases sensitivity to cisplatin (107). Thus, accumulated studies reveal that knockdown of mutant p53 by siRNAs or shRNAs reduces malignant properties of cancer cells. However, siRNAs for p53 used in aforementioned publications can also knockdown wild-type p53. Matinez et al. (108) first developed a *p53^*R248W*^*-specific siRNA which did not affect wild-type p53. Recently, our laboratory also developed siRNAs specific for p53^R175H^ and p53^R273H^, which did not alter wild-type p53 expression levels (unpublished). We successfully showed reactivation of wild-type p53 and reduced cell proliferation and migration, following transfection of these mutant-specific siRNAs in genetically engineered *p53* heterozygous cancer cell lines (HCT116^+/R248W^, SW48^+/R273H^) (unpublished). Thus, the extraordinary sequence specificity of siRNA makes it an attractive tool for targeted cancer therapies.

### Compounds That Affect Downstream Targets of Mutant p53

Another way to target oncogenic activity of mutant p53 is to reactivate tumor suppressive pathways that are inhibited by mutant p53 or to inhibit tumor-promoting pathways that are activated by mutant p53.

Reactivate transcriptional activity (RETRA) was identified as a compound that increased β-galactosidase activity in A431 (p53^R273H^) human epidermal carcinoma cells expressing a p53-resonsive promoter-driven β-galactosidase construct, through high-throughput screening of a chemical library comprising of 46,250 compounds (109). RETRA increases β-galactosidase activity only in cancer cells carrying mutant p53 (p53^R248Q^, p53^R280L^, and p53^G266E^) with increased mRNA expression of *p21* and *PUMA*, but fails to do so in cells wild type or null for p53. Interestingly, the effects of RETRA are nullified by knockdown of p73, but not knockdown of p53 and p63 (109). Indeed, RETRA inhibits the binding of p53^R273H^ with p73. RETRA also reduces A431 cell viability in a p73-dependent manner and reduces tumor growth in a xenograft model (109).

Statins are a class of compounds that inhibit 3-hydroxy-3-methylglutaryl coenzyme A (HMG-CoA) reductase in the mevalonate pathway and have been used in the clinic to treat hypercholesterolemia. HMG-CoA reductase is the rate-limiting step in cholesterol synthesis and also regulates prenylation/lipidation (farnesylation and geranyl-geranylation) of proteins. Prenylation facilitates attachment of target proteins to cell membranes which are involved in cellular adhesion, migration, and proliferation signaling (e.g., Rho, Rac, Cdc42, Ras) (110). Interestingly, knockdown of p53^R273H^ significantly reduces mRNA expression of multiple enzymes involved in the mevalonate pathway (16). Both knockdown of mutant p53 and inhibition of protein prenylation by statins or other compounds result in impaired growth of breast cancer cells in 3D culture (16). Mechanistically, mutant p53 binds to and activates SREBP, crucial transcription factors that regulate transcription of several enzymes involved in the mevalonate pathway, leading to enhanced prenylation of proteins associated with cancer progression and activation of prenylated proteins in breast cancer cells; hence, inhibition of protein prenylation by statins leads to reduced malignancy of human breast cancer cells (16). Importantly, the presence of p53 mutation correlates with high expression of sterol biosynthesis genes in human breast tumors (16). Additionally, since nuclear localization and activation of the YAP and TAZ proto-oncogenes are regulated by prenylation and activation of Rho GTPases, statins could also suppress progression of mutant p53-expressing tumors by inhibiting YAP/TAZ activation by reducing protein prenylation of Rho GTPases, which is promoted by SREBP and its cofactor mutant p53 (111).

### Compounds That Induce Synthetic Lethality

Synthetic lethality is generally used for the condition where a mutation in a gene is not lethal by itself, but its combination with a drug or other gene mutations leads to cell death (112). Since over 50% of human cancers have mutations in the *p53* gene, p53 mutations become attractive targets for inducing synthetic lethality in tumors. In this regard, compounds that induce synthetic lethality to mutant p53 should selectively kill cancer cells expressing mutant p53 without affecting normal cells carrying wild-type p53. One compound that induces synthetic lethality to mutant p53 is UCN01, a protein kinase C inhibitor and a potent blocker of G2/M checkpoint of the cell cycle. Treatment of CA46 (p53^R248Q^) and HT29 (p53^R273H^) with UNO01 abrogates γ-irradiation-induced G2/M arrest of the cell cycle and increases cytotoxicity (113). UCN01 also enhances cisplatin-induced cell death in MCF7-expressing human papillomavirus type-16 E6 (MCF7/E6) with little effect on parental MCF7 cells having functional p53 (113). Another compound that is synthetic lethal to mutant p53 is BI-2536, an inhibiter of polo-like kinase 1 (PLK1), an enzyme that controls G2/M checkpoint. Inhibition of PLK1 by BI-2536 significantly enhances cytotoxic effect of ionizing radiation in DLD-1 (p53^S241F^) and p53^R248W^-overexpressing HCT116 cells, but it does not do so in parental HCT116 (wild-type p53) cells (114). Similar effects are observed with PD0166285, an inhibitor of Wee1 kinase that regulates G2/M checkpoint. PD0166285 sensitizes cancer cells (HT29: p53^R273H^ and E6-overexpressing PA-1: wild-type p53) to radiation-induced death, whereas this effect is not detected in parental PA-1 cells (115). However, the observed synthetic lethality to mutant p53 is likely not dependent on oncogenic GOF activity of mutant p53, but rather dependent on loss of wild-type p53 activity. Identification of synthetic lethality to mutant p53 alone, but not p53-null, could improve our understanding of oncogenic GOF activity of mutant p53 and contribute to the development of new compounds that target cancer cells carrying mutant p53.

## Summary and Future Directions

Accumulated evidence has proven that small-molecule compounds can restore the transcriptional activity of mutant p53 or specifically deplete mutant p53. These compounds are expected to efficiently inhibit tumor growth with minimal effects on normal tissues. Several compounds listed in Tables 1 and 2 are already in clinical trials. Within p53 reactivators, PRIMA-1^MET^ (also known as APR-246) is the only drug under clinical trials (51, 52). On the other hand, amongst the compounds that deplete mutant p53, Hsp90 inhibitors (81–83), HDAC inhibitors (116–118), ATO (119–121), and DSF (92, 93, 122) are under clinical trials for cancer therapy. However, it is not completely understood whether these p53 reactivators and mutant p53-depleting compounds have mechanisms of action on proteins or pathways other than mutant p53, if they have an impact on all p53 mutants or specific mutant types and their underlying mechanisms. Also, it would be important to determine any synergistic or additive effects of these compounds with conventional chemotherapy drugs on cancer cell survival and proliferation. Further studies to solve these concerns would help improving the efficacy and specificity of these compounds.

In order to better understand the mechanisms of action of compounds that target mutant p53, it is important to determine whether they can directly bind with mutant p53 or proteins involved in the process of mutant p53 reactivation or depletion. There are several *in vitro* methodologies to determine compound–protein interaction, including Biacore assays, mass spectrometry-based approaches, and drug affinity responsive target stability (DARTS) assays (123–125). On the other hand, *in vivo* analysis is limited. Recently, Jafari et al. (126) reported a cell-based drug–protein interaction assay, called cellular thermal shift assay (CETSA). Investigating interactions between a compound and a specific protein in cells would significantly improve our understanding of how efficiently and specifically the compound alters intracellular activity.

Biological effects of compounds that restore p53 activity are robust, since mutant p53 is usually accumulated in cancer cells, and hence these compounds have ample substrates to restore the p53 activity. On the other hand, compounds that deplete mutant p53 may not be as robust as those restoring the p53 activity; however, as mentioned in Section “Introduction,” survival and growth of cancer cells are frequently dependent on mutant p53 (oncogene addiction) (13, 14). Thus, simply depleting mutant p53 in tumors is likely sufficient to reduce tumor malignancy. Especially, when tumor cells retain the wild-type *p53* allele, compounds depleting mutant p53 alone may be even more effective, since they could reactivate wild-type p53 by releasing from DN activity of mutant p53. These mutant p53-depleting compounds could also be used for prevention of tumorigenesis when patients inherently carry mutant p53, such as in the case of human tumor-prone disease Li–Fraumeni syndrome (LFS). Over 70% of LFS patients have p53 mutations in their germlines (127), but mutant p53 is not always stable and accumulates mainly in tumors (10). A compound that depletes only accumulated mutant p53 in tumors could reduce risk of tumor development in LFS patients. The approach of mutant p53-specific knockdown by siRNAs or DNA oligomers could cause similar outcomes to those by mutant p53-depleting compounds. However, the major hurdle of this approach is efficient delivery of these oligomers to all cancer cells, which need to be addressed prior to their consideration for clinical trials (103).

Other than compounds summarized in this review article, compounds that target inhibitors of p53, such as MDM2 and MDM4, are also powerful to reactivate p53 and are summarized in other review articles (128, 129). Also, induction of synthetic lethality for mutant p53 is another specific approach for cancer cells expressing mutant p53 (128, 130–132). Inhibitors of proteins associated with G2/M arrest of the cell cycle and mitotic checkpoint appear to cause mitotic catastrophe in cancer cells lacking wild-type p53 activity.

In summary, discovering efficient and safe compounds that specifically target mutant p53 remains challenging. Hence, it is crucial to further understand how mutant p53 induces oncogenic function, to elucidate the exact mechanisms of mutant p53 stabilization or degradation in tumors, and to identify mutant p53-specific downstream signaling pathways or binding partners. The battle with cancer is unexpectedly taking longer, with cancers being wily enough to escape from current available treatments and develop novel ways of surviving. Given that several mutant p53-targeting drugs are already undergoing clinical trials, the goal toward establishment of therapies to cure mutant p53-carrying cancers may be just on the horizon.

## Author Contributions

AP and TI wrote the manuscript.

## Conflict of Interest Statement

The authors declare that the research was conducted in the absence of any commercial or financial relationships that could be construed as a potential conflict of interest.
